# Cerebral venous sinus thrombosis associated with thrombocytopenia post-vaccination for COVID-19

**DOI:** 10.1186/s13054-021-03572-y

**Published:** 2021-04-12

**Authors:** Gian Paolo Castelli, Claudio Pognani, Carlo Sozzi, Massimo Franchini, Luigi Vivona

**Affiliations:** 1 Department of Anesthesiology and Intensive Care, “Carlo Poma” Hospital, ASST Mantova, Via Lago Paiolo, 10, 46100 Mantova, Italy; 2 Neuroradiology, “Carlo Poma” Hospital, ASST Mantova, Mantova, Italy; 3 Department of Hematology and Transfusion Medicine, “Carlo Poma” Hospital, ASST Mantova, Mantova, Italy; 4 Department of Pathophysiology and Transplantation, University of Milan, Milan, Italy

## Introduction

Cerebral venous sinus thrombosis (CVST) is a rare form of stroke generally occurring in younger patients (typically < 50 years old), predominantly women and amounting to 0.5–1% of all strokes. Incidence is about 5–16 cases per 1 million people per year [1–3].

CVST has been reported in COVID-19 patients associated with thrombocytopenia [4, 5]; on March 15, 2021, the Paul Ehrlich Institut (Federal Institute for vaccines and biomedicines) reported CVST in seven patients 20–50 years old after vaccination with COVID-19 vaccine AstraZeneca.

We report about a previously healthy 50-year-old Caucasian man admitted to the city hospital of Mantua on March 15, 2021, with severe headache during the previous four days, slight deviation of the right buccal rim, loss of strength in the right lower limb, unstable walking and slight visual impairment. On March 4, 2021, he had received the first dose of the anti-COVID-19 AstraZeneca vaccine with no immediate adverse reaction.

**Fig.1:**
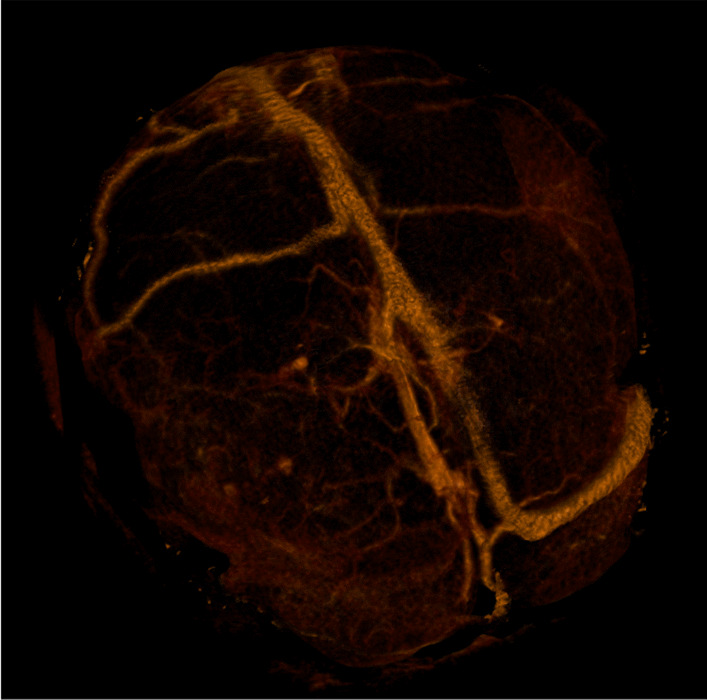
3D reconstruction with lack of opacification of the left transverse sinus

On examination, he was apyretic, arterial pressure 150/80 mmHg and heart rate 80/min, SpO_2_ 99% in room air, GCS 15, pain numerical rating scale 8/10. Laboratory blood tests showed marked abnormalities in blood coagulation (Table 1). The patient was a volunteer blood donor, and previous routine blood tests had repeatedly reported normal platelet counts. SARS-CoV-2 Buffer (RT-PCR) and Anti-SARS-CoV-2 Antibody Search were negative. A brain CT scan showed intra-parenchymal haemorrhage in the left hemisphere, while CT angiography showed multiple bleeding spots within the parenchymal haemorrhage and lack of opacification of the left transverse and sigmoid sinuses, suggesting thrombosis of the venous sinuses (Fig. 1). Four hours after admission, the patient had deteriorated to GCS 8, right hemiplegia, localization of the painful stimulus to the left, no execution of orders nor verbal production. He showed isochoric, isocyclic pupils and vomiting. The patient was transferred to the intensive care unit (ICU), and a thromboelastogram (TEG6S, Haemonetics) showed a prolonged reaction time, a decreased platelet function and lack of fibrinogen, with marked reduction of maximum amplitude of the clot; fibrinogen concentrate (10 g total) and platelet (4 units total) were administered.

**Table 1:** Abnormal laboratory parameters

Parameter	Patient’s values	Normal values
Platelets (10^9^/L)	20	150–400
Fibrinogen (mg/dL)	98	150–450
D-dimer (ng/mL)	> 10,000	< 500
C reactive protein (mg/L)	17.6	< 5
Coagulation factor XIII (%)	35	70–150
Methylenetetrahydrofolate reductase (MTHFR) mutation (C677T)	Heterozygous	Absent
Homocysteine (μmol/L)	16.7	< 12
Folic acid (ng/mL)	0.9	3.9–26.8

Six hours after ICU admission, the patient had become medium mydriatic: he was intubated, sedated and paralysed and underwent a second CT scan, which documented the increase in the haemorrhagic focus, the initial transtentorial herniation of the left temporal uncus and a shift of the midline to the right. The patient underwent a bilateral decompressive craniectomy, which confirmed diffuse thrombosis of the cortical veins. Upon returning from the operating room, a subsequent TEG6S showed normalization of the clot formation rate and reduced clot strength, with PLT had fall back to 15,000/mcL.

Antiplatelet antibodies were negative, while a heterozygous state for the MTHFR thrombophilic mutation with increased levels of hyperhomocysteine and concomitant folate deficiency were reported post-mortem.

Haemodynamic instability, mydriasis and lack of intra-cerebral blood flow at CT angiography led to the diagnosis of brain death, approximately 48 h after admission to the hospital.

## Discussion

SARS-CoV-2 infection has been associated with hypercoagulability, with a high incidence of venous thromboembolism including pulmonary embolism and deep vein thrombosis. Physicians should also be alert for signs and symptoms related to thromboembolism when they occur in patients who have recently been vaccinated with the COVID-19 AstraZeneca vaccine.

## Data Availability

The dataset used and/or analysed during the current study is available from the corresponding author on reasonable request.
